# Fretting Fatigue Analysis of Additively Manufactured Blade Root Made of Intermetallic Ti-48Al-2Cr-2Nb Alloy at High Temperature

**DOI:** 10.3390/ma11071052

**Published:** 2018-06-21

**Authors:** Mario Lavella, Daniele Botto

**Affiliations:** Politecnico di Torino Department of Mechanical and Aerospace Engineering, Corso Duca degli Abruzzi, 24, 10129 Torino, Italy; daniele.botto@polito.it

**Keywords:** fretting-fatigue, contact, additive manufacturing, intermetallic, titanium aluminum alloy

## Abstract

Slots in the disk of aircraft turbines restrain the centrifugal load of blades. Contact surfaces between the blade root and the disk slot undergo high contact pressure and relative displacement that is the typical condition in which fretting occurs. The load level ranges from zero to the maximum during take-off. This cycle is repeated for each mission. In this paper, a fretting fatigue analysis of additively manufactured blades is presented. Blades are made of an intermetallic alloy γTiAl. Fretting fatigue experiments were performed at a frequency of 0.5 Hz and at a temperature of 640 °C to match the operating condition of real blades. The minimum load was fixed at 0.5 KN and three maximum loads were applied, namely 16, 18 and 20 kN. Both an analytical and a two-dimensional finite element model were used to evaluate the state of stress at the contact interfaces. The results of the analytical model showed good agreement with the numerical model. Experiments showed that cracks nucleate where the analytical model predicts the maximum contact pressure and the numerical model predicts the maximum equivalent stress. A parametric analysis performed with the analytical model indicates that there exists an optimum geometry to minimize the contact pressure. Tests showed that the component life changed dramatically with the maximum load variation. Optical topography and scanning electron microscopy (SEM) analysis reveals information about the damage mechanism.

## 1. Introduction

There is a continuous demand for increasing the efficiency of modern aircraft engines due to the need for economical and environmentally friendly air transport. One solution is to reduce the mass of engine components. The mass reduction of rotating components like blades also decreases the centrifugal loads and then the contact forces needed on the disk to restrain the blade. The disk thickness can be reduced accordingly, with beneficial effects on the mass of the whole engine. This leads designers to increase the use of lighter materials. From this point of view, blades made of an intermetallic alloy γTiAl to replace the blades made of nickel-based superalloy seem to be an attractive alternative. Titanium aluminide γTiAl has a density of about 4200 kg/m^3^, and it is 50% lighter than the nickel-based alloys currently used for low-pressure turbine blades. Blades made from this material can reduce the weight of the entire low-pressure turbine by 20%. Moreover, this alloy shows outstanding strength performance in the temperature range between 600 °C and 800 °C. The specific stiffness *E/ρ* (*E* and *ρ* being the Young’s modulus and density respectively), the specific strength σ*_y_/ρ* (σ*_y_* being the yield stress), and the creep resistance at 700 °C of γTiAl are higher compared with the superalloy Inconel.

At the component level, both the material properties and the production processes are of paramount importance to obtain appropriate fatigue behavior [1,2]. Regarding the fatigue strength of the attachment, more aspects need to be evaluated [3]. There are two main types of cyclical load, one generated by the blade vibration and another caused by the blade centrifugal load.

High-frequency vibrations induce dynamical loads that could trigger high-cycles fatigue (HCF) failures if not promptly damped. Sliding of the contact surfaces of the attachment introduces additional damping that is beneficial to reduce the blade vibrations [4]. The effect of friction damping at the blade root on the forced response of rotating bladed disks is not negligible and it is widely studied in the literature [5,6,7].

The stress variation induced in the attachment by the blade centrifugal force is related to changes in the rotational regime of the aerospace engine and is a low frequency phenomenon. The blade centrifugal force ranges from zero to its maximum—during take-off—with minor variation throughout the flight. Few loading cycles associated to a high level of stress are expected for each mission. Consequently, a low-cycles fatigue (LCF) analysis is necessary to assess the blade attachment strength.

Both these loads cause relative displacements between the contact surfaces of the attachment. This is the typical condition in which fretting occurs, as fretting is the process of damage by fatigue, wear and corrosion that results when the mating surfaces undergo low-amplitude relative reciprocating sliding. Fretting fatigue at the blade root is one of the most important causes of in-service damage [8]. Fretting is widely studied and many papers containing experimental results and damage models can be found in the literature. Usually, experiments are performed by using test rigs that simulate the contact of two mating surfaces whose geometry is as close as possible to the real contact. Tests can be performed in load or displacement-controlled conditions. Most of these experiments, such as [9], are based on the study of conformal (flat on flat) or non-conformal (sphere or cylinder on flat) contact geometries. A few fretting studies [10,11] focus on the specific dovetail geometry of the blade root.

This research is focused on the evaluation of the fretting fatigue properties of additively manufactured blade roots made of intermetallic alloy Ti-48Al-2Cr-2Nb. Investment or spin casting are usually used to manufacture TiAl parts. However, this material has a very high Poisson’s ratio and can become brittle and prone to cracks as it cools. Electron beam melting (EBM) minimizes these problems. In this process, a localized melting of metal powder is obtained by a powerful electron beam. Electrons are generated in a gun accelerated using high potential and focused on the position defined by a computer-aided design (CAD) model by means of a lenses system. Powder hoppers are used to feed the powder onto the build table where the electron beam is focused. This table is lowered in order to builds each successive layer. In other words, each layer is built on a focus plan where the electron beam moves in x–y directions in order to scan the whole layer. A vacuum system enables the maintenance of a low pressure (the magnitude is 10^−5^ mbar) during the layers’ building. A description of EBM compared with selective laser melting is reported by [12]. Moreover, EBM builds blades from layers of powder that are more than four times thicker than those used in more conventional additive manufacturing methods. This technology is expensive but the weight and fuel consumption savings makes EBM very competitive with other methods.

A few data are available in the literature about the mechanical properties of this alloy. Reference [13] reports the tensile and microstructure properties of this γTiAl alloy obtained by additive manufacturing. Paper [14] reports the LCF properties of the alloy Ti-48Al-2Cr-2Nb obtained by investment casting. For the same alloy, a fretting wear analysis was also performed, using specimens produced with a casting process, and published in [15]. Also, a characterization of a welded joint of an additive manufactured γTiAl alloy-steel can be found in [16].

The objective of this paper is to fill in gaps in knowledge about the fretting fatigue of blades in the alloy Ti-48Al-2Cr-2Nb obtained by additive manufacturing. Dummy blades with dovetail attachments were additively manufactured through EBM. A specific dummy disk sector made of the nickel-based superalloy Inconel 718 with a dovetail type slot was purposefully developed. Moreover, an induction system was designed to perform tests at high temperature.

The contact surface morphology was measured with a focus variation optical system. The damage mechanism was investigated by observing the fracture surface with scanning electron microscopy (SEM). Moreover, analytical and finite element (FE) models were used to compute the stress distribution within the contact region and its surroundings. The analytical model indicates a methodology for mitigating the crack nucleation by means of an optimum radius of the end contact disk surface. The experimental results reveal a substantial brittle crack propagation started at the contact with the external end of the disk contact surface i.e., at the edge of the bedding. Moreover, the component life changed dramatically with a small variation of the maximum load. The design criteria should be upgraded accordingly.

## 2. Materials and Methods 

### 2.1. Test Rig

**Figure 1:**
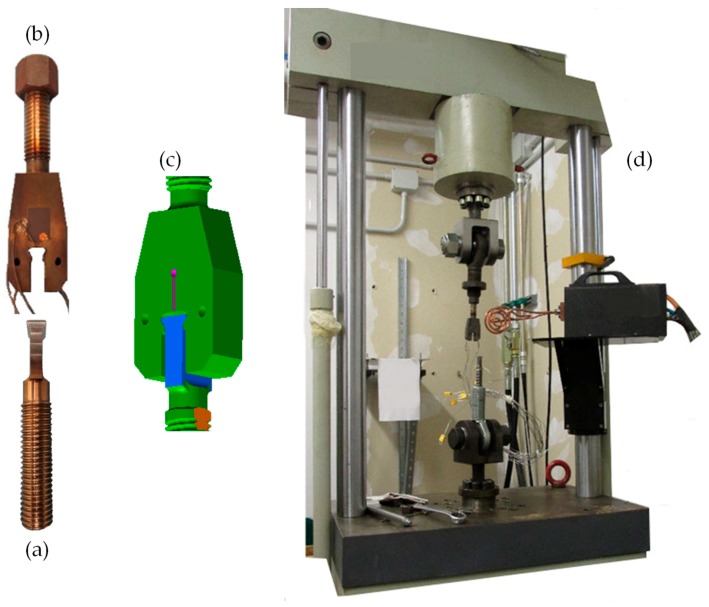
Test rig: (**a**) model of blade root; (**b**) model of disk cavity; (**c**) assembly of disk and blade root; (**d**) overall view.

Experiments were performed with the specific test rig depicted in Figure 1. A uniaxial testing machine provided the axial force to simulate the centrifugal load on the blade. The dummy blade specimen is shown in Figure 1a. All specimens were made of intermetallic alloy Ti-48Al-2Cr-2Nb, manufactured through EBM. These dummies were specifically manufacture for this experiment with the same process that will be used for the real blade. It can be reasonably assumed that the microstructure of the dummy and real blade was the same. Also, the attachment dimensions in the dummy are the same as the real blades. The blade root was manufactured in the form of dovetail. Regarding wear mitigation, a Ni 22Fe 16Cr 1.5Si Sulzer Metco® coating with a thickness of 200 μm was applied by air plasma spray on the contact surfaces of dummy blades. To diminish the friction coefficient the coated surfaces was painted using a Surf-Kote® LOB-1800-G with a thickness 12–25 μm. The average roughness of the contact surfaces, measured after deposition, was R_a_ = 7 μm. The airfoil of the real blade was replaced by a M20 thread needed to assemble the dummy blade into the test rig.

The dummy disk sector is shown in Figure 1b. It was made of Inconel 718 by milling with the same dimensional and geometric tolerances as real disks. Slots on real disks are usually broached but this discrepancy in terms of manufacturing process do not affect the fretting results, provided the roughness on the contact surface is the same. The maximum roughness of the contact surfaces on the dummy disk slot was R_a_ = 1.6 μm. The contact interfaces between dummies blade and disk sector are rectangular with a size of 2mm × 20 mm and symmetrically located. A M20 thread was also machined on the back of the dummy sector to assemble the dummy disk in the test rig. Figure 1c shows the blade/disk assembly, and Figure 1d shows the overall view of the experimental setup.

**Figure 2:**
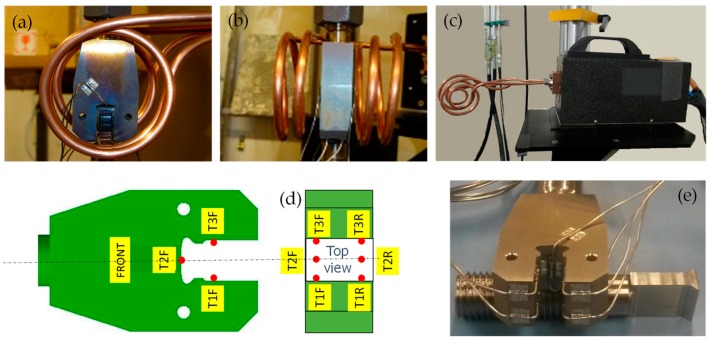
Heating coil setup front view (**a**) and side view (**b**); induction head with coil (**c**); sketch of thermocouple placement (**d**,**e**).

The dummy disk was tightened into a fixture connected to the load cell on the testing machine. Likewise, the dummy blade was tightened into a similar fixture connected to the hydraulic actuator. The stroke of the hydraulic actuator was measured by a linear variable displacement transducer (LVDT). The fixtures included a spherical hinge to avoid additional moments on the dummies when the axial force was applied, see Figure 1d. The attachment was heated with an induction furnace. The heating coil setup is shown in Figure 2a,b. Different coils were tested and evaluated in terms of uniformity of the temperature distribution. Figure 2c reports the induction head and the coil used during the tests. This in-house made coil had a two spirals form facing the contact area. Tests were performed at 640 °C. Temperature was controlled by means of six thermocouples type K placed on both sides of the disk slot as shown in Figure 2d,e. The maximum difference in temperatures among thermocouples observed during the experiments was about 10 °C.

Fretting fatigue experiments were performed at a frequency of 0.5 Hz. To keep the slot and the blade root dummy continuously in contact, a minimum load of 0.5 kN was applied during all tests. Tests were performed with three maximum loads, namely 16, 18 and 20 kN. These loads were representative of the real working conditions of the blade under investigation. At the end of the test, the contact area and fracture surfaces were measured by optical topography and observed with SEM.

### 2.2. Analytical Model

**Figure 3:**
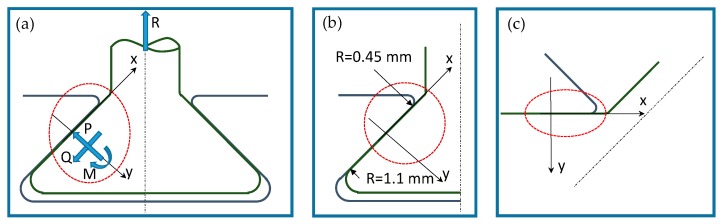
Contact geometry: (**a**) blade root; (**b**) contact surfaces of one side; (**c**) rotated half contact surfaces of one side.

**Figure 4:**
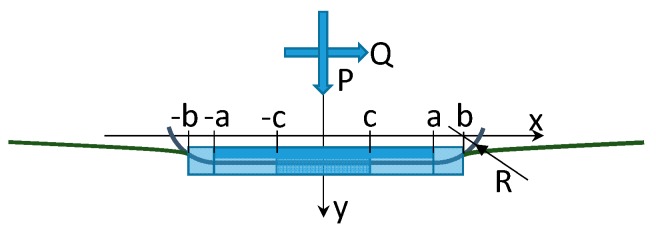
Rounded punch geometry.

Beforehand, the state of stress in the contact region of the attachment, Figure 3, was studied with the analytical model of a flat punch with rounded edges. The two states of stress are equivalent if a proper equivalent radius is chosen and the stress distribution is symmetrical. The general dovetail geometry reported in Figure 3a shows a reference system (x, y) located at the center of the contact area. Forces P and Q are the normal and tangential forces applied at the contact area center. M is the moment due to non-symmetrical pressure distribution. The dovetail geometry can be reduced to the geometry of Figure 3c, in which the minimum equivalent radius is used. Figure 4 shows details of the contact geometry of the punch. Due to symmetrical stress distribution, the moment M is null.

The punch has a flat base of width 2*a* and two arcs whose radius is R (fillet radius). As reported in [17] the normal pressure distribution in a flat punch with rounded edges is:

(1) $$p\left(\varphi \right)=P\cdot b\cdot f\left(\varphi ,\varphi_{0}\right)$$

where *b* is the half-width of the contact area, *P* is the normal load per unit of length. See Figure 4 for details. The auxiliary function *f*(*ϕ*, *ϕ*_0_) depends on the auxiliary angle,

(2) sin(φ)=x/b

The auxiliary angle *ϕ*_0_ gives the size of the contact area and can be found solving the implicit equation:

(3) $$\frac{4PR}{a^{2}E^{*}}=\frac{\pi -2\varphi_{0}}{2\sin^{2}\left(\varphi_{0}\right)}.\cot\left(\varphi_{0}\right)$$

where sin(*ϕ*_0_) = *a*/*b*, *a* is the half-width of the contact area without normal load, (see Figure 4) 1/E* = (1 − ν_1_^2^*)/E*_1_ *+ (1 − ν*_2_^2^*)/E*_2_ where *E*_1_, *E*_2_ are the Young’s moduli and *ν*_1_, *ν*_2_ the Poisson ratios of two materials. According to [17] the tangential stress distribution *q*(*x*) is

(4) $$q\left(x\right)=\mu \cdot \left(p\left(x\right)-p^{*}\left(x\right)\right)=\mu \cdot p\left(x\right)-q^{*}\left(x\right)$$

namely the difference between the actual pressure distribution, *p*(*x*) and the pressure distribution for a smaller contact area p*(*x*), multiplied by the coefficient of friction *μ*. The expression for q* in Equation (4) is similar to that given for *p* in Equation (1).

(5) $$q^{*}\left(\vartheta \right)=\frac{\mu P-Q}{c}\cdot f\left(\vartheta ,\vartheta_{0}\right)$$

*Q* is the tangential load per unit of length and *c* is the half length of the transition between the stick and slip region in the contact area (see Figure 4). This transition can be found solving the equation:

(6) $$\frac{4PR}{a^{2}E^{*}}\left(1-\frac{Q}{\mu P}\right)=\frac{\pi -2\vartheta }{2\sin^{2}\left(\vartheta \right)}.\cot\left(\vartheta_{0}\right)$$

where sin(*θ*_0_) = *a*/*c*. More details about the auxiliary function are given in [18]. Equations (1) and (4) give the normal and tangential stresses on the contact surface. The state of stress beneath the surface can be determined with the potential function of Muskelishvili [19] as reported in [20]. The friction coefficient *μ* was measured using test rigs and procedures described in [21,22,23,24].

### 2.3. Numerical Model

The analytical model presented in the previous section gives very accurate results, in terms of state of stress, for the punch geometry. When this model is applied to the real geometry of the dovetail it does not consider the flexibility of the whole attachment and the results are less accurate. Therefore, a numerical model is needed.

**Figure 5:**
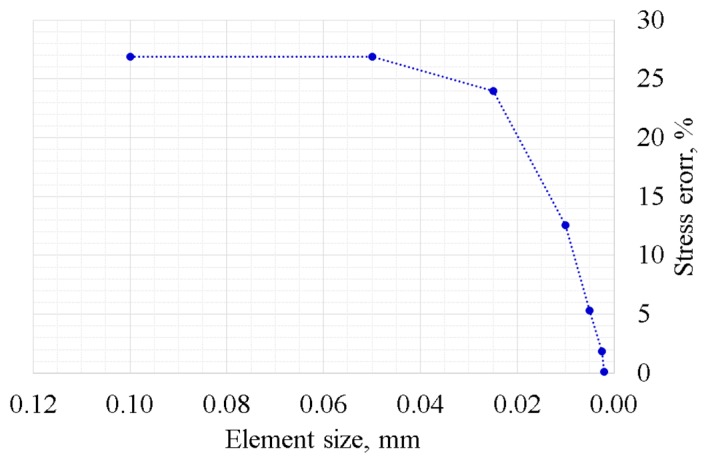
Error in normal contact stress between the finite element (FE) and analytical models.

A two-dimensional FE model was developed with standard commercial software within the hypothesis of plane strain. As the size of the mesh is crucial for a reliable analysis, the normal stress computed with the FE model of the rounded edges punch was compared with the stress given by the analytical solution on the same geometry. Figure 5 shows the results of the comparison, in terms of relative error between the stress computed with the different models. A mesh with element size 0.005 mm was considered fine enough for the contact problem simulation. The attachment was meshed with a quadratic tetrahedron. The augmented Lagrangian formulation was used to solve the contact problem.

## 3. Results and Discussion

**Figure 6:**
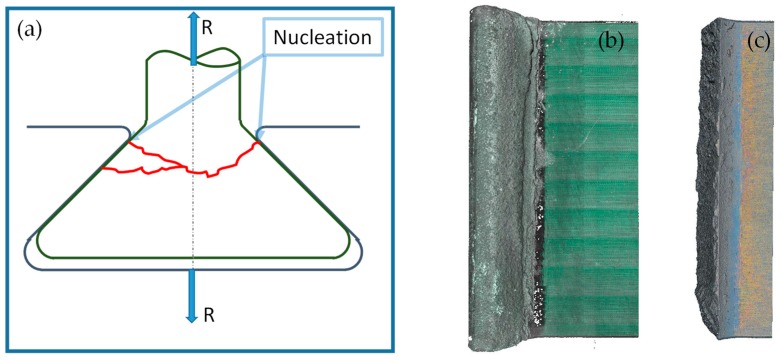
Crack evolution and optical topography of specimen: (**a**) typical fracture nucleation and propagation; (**b**) before fretting fatigue test; (**c**) fretting fatigue test.

The results of the low-cycle fatigue experiment are reported in Table 1. In this type of application, 30,000 cycles is the expected life, thus experiment number 3 was stopped before failure (runout). Figure 6a shows a typical nucleation and propagation fracture. Figure 6b,c shows the optical topography of a specimen before and after the fretting fatigue test. It can be see that failure occurs on the blade close to the external end of the disk slot.

**Figure 7:**
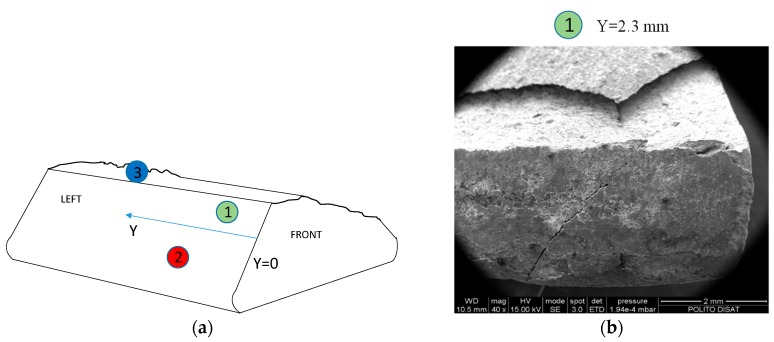

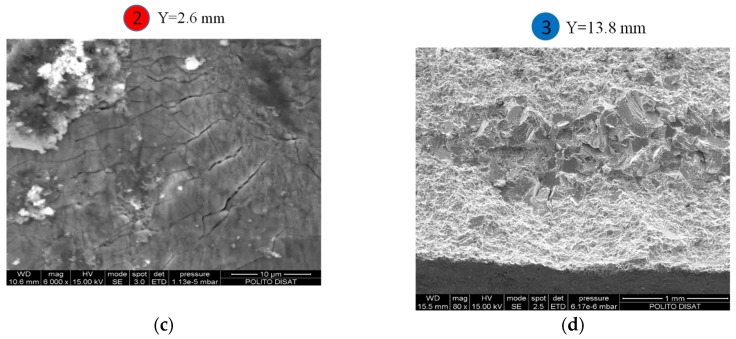
Scanning electron microscope (SEM) micrographs of test # 2, Y indicate the distance from the front surface: (**a**) position of SEM micrographs; (**b**,**c**) contact left surface; (**d**) fracture surface.

The experimental results underline that a small difference in load (4 kN, 20% of initial value) corresponded to a difference of fatigue life of about two orders of magnitude. This could imply that the fracture propagation is fast and then a brittle behavior is expected. In a gross slip regime, cracks are usually removed by wear at an early stage, particularly in ductile materials. In the observed failures, the damage mechanism started first with fretting wear of the coating. Fretting cracks on the coating are visible in Figure 7c. Due to these cracks, the coating does not mitigate tangential stresses of the contact surfaces. Thus, the fretting cracks on the coating are the points where there is more probably crack nucleation on bulk material of the blade. The thickness of the coating was 100 μm at the failure of specimen 1 (414 cycles), while it was 50 μm at the failure of specimen 2 (13,814 cycles). Due to the brittleness of the coating and bulk, the crack propagated at a higher velocity than the wear effect, even if the contact took place in the gross slip regime. The contact regime (mixed stick-slip or gross slip) can be assessed by observing the contact surfaces with SEM and by computing the relative displacement with the numerical model. SEM analysis of regions indicated by points 1, 2 and 3 in Figure 7a are reported in Figure 7b–d. Micrographs were taken on both contact and fracture surfaces. Figure 7b did not show stick areas on the contact surfaces, thus a gross slip regime is a reasonable hypothesis. This experimental evidence is consistent with the relative displacement between the contact surfaces computed with the FE numerical model. Moreover, there were not clearly visible wear grooves in the bulk material. Substantially, there were no grooves beneath the coating. Thus, it can be deduced that the crack propagation is faster than the wear process even if the contact surfaces undergo a gross slip regime. Figure 7b also shows an evident crack on the contact surfaces. Figure 7c shows typical brittle fretting cracks. Figure 7d shows a brittle fracture surface with intergranular and transgranular crack propagation.

**Figure 8:**
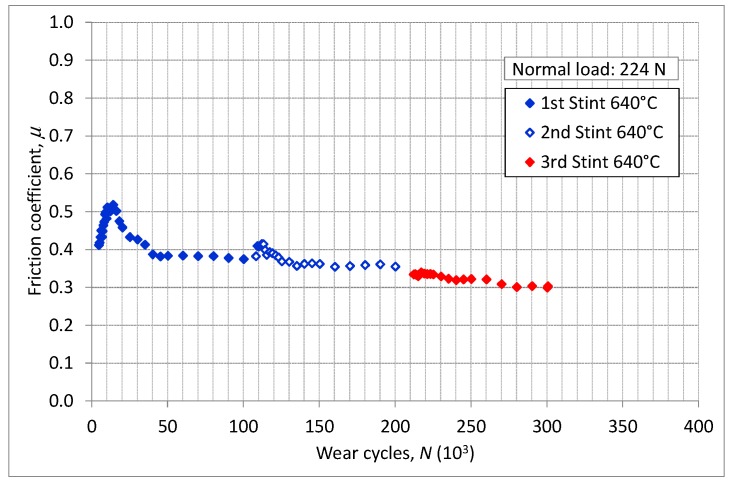
Friction coefficient measure with test rig flat on flat surface [23,24].

Different test rigs [21,23,24] were used to measure the friction coefficient. Specimens were covered with the same coating—applied with the same technological process—of the dummy blade. The contact surfaces of two specimens were provided in the same roughness condition as dummies. Experimental procedures and methodologies used are reported in [22]. Figure 8 shows the friction coefficient measured with flat-on-flat contact surfaces using the test rig described in [23,24]. The diagram shows the friction coefficient as a function of wear cycles. Results were recorded during three different measurement sessions, namely the 1st, 2nd and 3rd Stints. Between two stints, specimens were removed and cleaned. Discontinuity in the friction coefficient between two consecutive stints are due to the debris entrapped between the mating surfaces as explained in [21].

**Figure 9:**
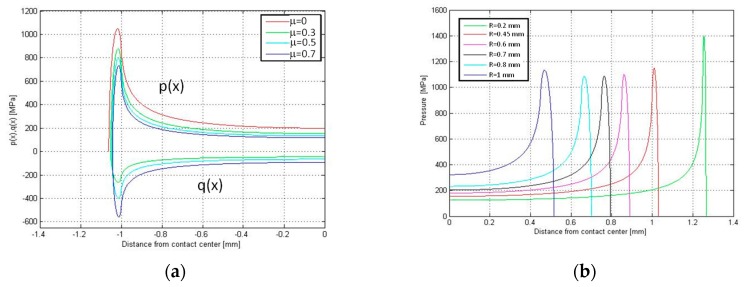
Analytical model of test # 2: (**a**) contact normal (p) and tangential (q) pressure for different friction coefficients; (**b**) normal contact pressure as a function radius of the external end of the disk slot.

The state of stress was computed first on a rounded punch, as shown in Figure 4. This computation was performed with the analytical the model at the maximum load of 20 kN. The rounded punch is composed of a flat base of width 2*a* and two arcs whose radius is R (fillet radius). The rounded punch is symmetrical, and so only half of the contact region is depicted in Figure 9. Figure 9a illustrates the distribution of normal (*p*(*x*)) and tangential (*q*(*x*)) contact stress along the contact line for different values of the friction coefficients *μ*. The tangential stress q has been determined in full sliding conditions. The fillet radius is R = 0.45 mm and corresponds to the outer radius at the edge of the bedding in the dovetail. Figure 9a shows that the contact pressure increases dramatically at the end of the contact where the fillet connects to the flat base. Figure 9b reports the normal pressure along the contact line for different fillet radii R. This analysis was made assuming a constant overall dimension of the punch. In other words, the distance between the endpoints of the two arcs, namely 2·(a + R), remains constant. Figure 9b shows that there is an optimum radius R = 0.7 mm for which the contact pressure reaches a minimum.

That means there is a balance between the beneficial effect of increasing the fillet radius and the detrimental effect of reducing the width of the flat base.

**Figure 10:**
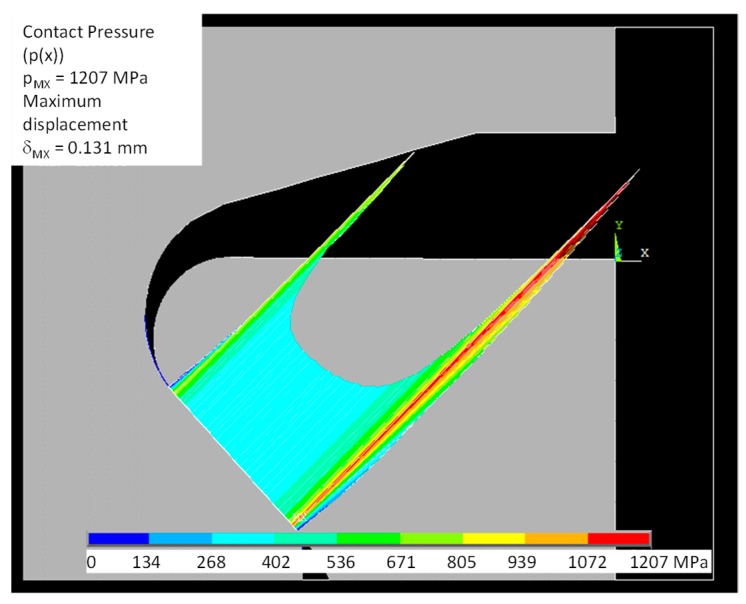
FEM model of test # 2: contact pressure.

Figure 10 reports the normal contact stress calculated by the FE model. It can be observed that the higher peak contact stresses are located at the lower radius, i.e., the outer edge of the bedding. The effect of the different radii (inner and outer) is quantified. The higher peak was also predicted with the analytical model and there is a good agreement between the numerical and analytical models. The peak contact stresses is located at outer edge of the bedding and its value (1206 MPa) is very near to the prediction of the analytical model (about 1150 MPa), see Figure 9b at R = 0.45 mm. The difference between the numerical and analytical models is mainly due to the compliance of the disk (the analytical model neglects this effect). Moreover, these pressure distributions are even coherent with results obtained by the model [25] based on a different methodological approach.

**Figure 11:**
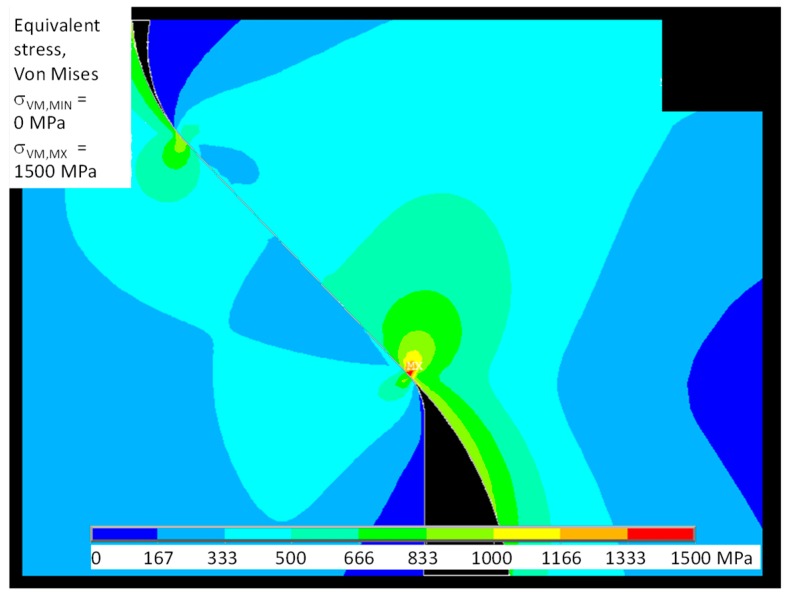
FE model of test # 2: Von Mises stress.

**Figure 12:**
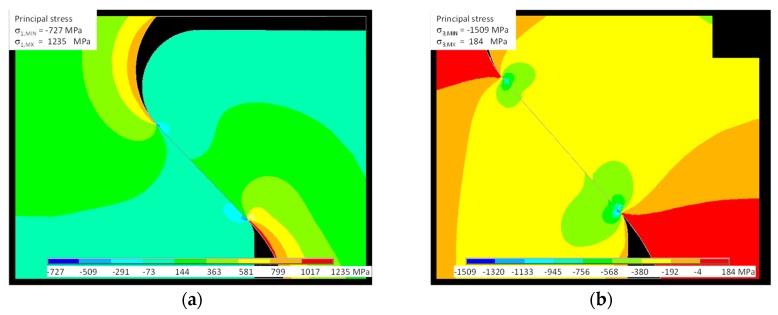
FE model of test # 2: (**a**) principal stress σ_1_; (**b**) principal stress σ_3_.

Figure 11 shows the spatial distribution of Von Mises stress; this failure criterion is important for analyzing the ductile behavior of the dummy disk (Inconel 718). Figure 12 shows the principal stress distribution σ_1_, σ_3_ more appropriate to the brittle behavior of the dummy blade (γTiAl). Figures also highlight how the maximum stress is located at the contact between the external end of the disk slot and the dummy blade root.

## 4. Conclusions

In this work, a preliminary study of fretting fatigue of an additively manufactured blade root made of intermetallic alloy Ti-48Al-2Cr-2Nb was undertaken.

The results, in terms of state of stress, given by the analytical model show good agreement with the FE model. Both models predict the maximum principal stress at the edge of the bedding closer to the blade shank. Experimental evidence shows that cracks leading to the final failure nucleate at that position.

The analytical model indicates that it is possible to find an optimum radius at the end of contact surfaces to minimize the state of stress. Consequently, the optimization criteria assume an important role in blade design. 

The experiment shows a brittle fatigue crack propagation. Moreover, the component life changes dramatically with the range of maximum load variation. This highlights also the necessity to use failure criteria appropriate to brittle materials in both static and fatigue analysis.

An indication of the damage mechanism was found. The role of coatings, in this case, seems to be very important for the endurance of the component. In contrast to the usual behavior in this material, fatigue cracks propagate also in the gross slip regime. An explanation for this could be that the crack propagation is fast and the wear process cannot remove cracks in the early stage.

To use blades made of intermetallic alloy Ti-48Al-2Cr-2Nb in actual turbines, it is fundamental to develop appropriate design criteria, failure criteria, and shape optimization of the blade root. Future research should be focused on the manufacturing process to reduce the brittleness of the material. This improvement in the material properties could facilitate many applications of this alloy in aero-engines. Moreover, a direct experimental comparison of components’ performances between conventional versus additively manufactured should be very useful. Even if the interest in additively manufactured components seems to justify that, a loss of performance is acceptable in comparison with the general advantage of the additive manufacturing process.

## Figures and Tables

**Table 1:** Results of tests at 640 °C.

Test #	Minimum Load, KN	Maximum Load, KN	Average Load, KN	Alternating Load, KN	Cycles to Failure
1	0.5	20	10.25	9.75	414
2	0.5	18	9.25	8.75	13,814
3	0.5	16	8.25	7.75	30,183 ^1^

^1^ Test stopped, no failure detected at 30,183 cycles.
